# Race and Disability Characteristics and Accommodation Disparities on the USMLE Step 1

**DOI:** 10.1001/jamanetworkopen.2025.34621

**Published:** 2025-09-30

**Authors:** Mytien Nguyen, Gabriel Abrams, Tiffany Hodgens, Raymond H. Curry, Michael H. Kim, Rylee Betchkal, Christine Low, Sharad Jain, Shami Tarlanov, William H. Eidtson, Caitlyn Coates, Charles Weiner, Tonya Fancher, Lisa M. Meeks

**Affiliations:** 1 Yale School of Medicine, New Haven, Connecticut; 2 University of Michigan Medical School, Ann Arbor; 3 University of California, Davis School of Medicine, Sacramento; 4 University of Illinois College of Medicine, Chicago; 5 University of Minnesota Medical School, Minneapolis; 6 Department of Counseling and Clinical Psychology, Teachers College Columbia University, New York, New York; 7 Icahn School of Medicine at Mount Sinai, New York, New York.; 8 Geisel School of Medicine, Dartmouth College, Hanover, New Hampshire; 9 Department of Psychiatry, Harvard South Shore, Boston, Massachusetts; 10 Law Office of Charles Weiner, Doylestown, Pennsylvania

## Abstract

**Importance:**

Ensuring access to accommodations on high-stakes examinations is crucial for ensuring equal opportunity for medical students with disabilities (MSWD).

**Objective:**

To examine the associations between gender, race and ethnicity, disability type, timing of disability diagnosis, and access to specialized disability resource professionals (DRPs) with accommodation requests and approvals on the US Medical Licensing Examination (USMLE) Step 1.

**Design, Setting, and Participants:**

A cross-sectional study of MSWD who graduated between 2020 and 2023 was conducted across 9 US MD-granting medical schools. Variables included deidentified student data (ie, gender, race and ethnicity, disability type, and timing of diagnosis, either before or after matriculation) and presence of specialized DRPs.

**Exposure:**

Diagnosis of a disability.

**Main Outcomes and Measures:**

The primary outcome was USMLE Step 1 accommodation request and approval. χ^2^, Fisher exact, and post hoc pairwise tests were used to assess the associations between gender, race and ethnicity, disability type, timing of diagnosis, presence of specialized DRPs, and accommodation requests and approval.

**Results:**

Among 295 MSWD in the cohort (189 female [64%]), 41 (15%) identified as Asian, 162 (59%) as White, and 70 (26%) as underrepresented in medicine (URiM; ie, American Indian, Alaska Native, Black or African American, Hispanic, or Native Hawaiian or Pacific Islander). One hundred five students (36%) reported a psychological disability, 167 (58%) received a diagnosis before medical school matriculation, and 120 (41%) requested Step 1 accommodations. A higher proportion of White students requested Step 1 accommodations than Asian students (74 White students [46%] compared with 8 Asian students [20%]). No difference in Step 1 accommodation request rate was found for URiM students compared with White students. Compared with medical students with psychological disability (31 students [30%]), a higher proportion of students with attention-deficit/hyperactivity disorder (36 students [50%]) and chronic health conditions (27 students [53%]) requested Step 1 accommodations. A higher proportion of students who received a diagnosis before medical school (88 students [53%]) requested Step 1 accommodations compared with those who received a diagnosis after medical school matriculation (30 students [25%]). Among the 97 students with known Step 1 accommodation approval status, 67 (69%) received accommodations. Step 1 accommodation approval rates were lowest for medical students with psychological disabilities (14 students [52%]) and attention-deficit/hyperactivity disorder (13 students [52%]) and those who received a diagnosis after matriculation (9 students [43%]). MSWD in schools with specialized DRP support had higher approval rates for Step 1 accommodation requests than MSWD in schools without specialized DRPs (49 students [78%] vs 18 students [53%]).

**Conclusions and Relevance:**

In this cross-sectional study of MSWDs, Asian MSWDs had lower USMLE Step 1 accommodation request rates. Students with psychological disabilities and those who received a diagnosis after matriculation had lower request and approval rates. The presence of specialized DRPs was associated with higher accommodation approval rate. Addressing disparities in USMLE Step 1 accommodations is critical for promoting equity and fostering a more representative health care workforce.

## Key Points

**Question:**

What is the association of gender, race, ethnicity, type and timing of disability diagnosis, and access to a specialized disability resource professional (DRP) with US Medical Licensing Examination Step 1 accommodations request and approval?

**Findings:**

In this cross-sectional study of 295 medical students with disabilities, the Step 1 accommodations request rate was significantly lower for Asian students and students with psychological disabilities. Accommodation approvals were less likely for those with psychological or attention-deficit/hyperactivity disorder diagnoses, diagnoses after medical school matriculation, and those from schools without specialized DRPs.

**Meaning:**

These findings highlight the disparate experiences of students requesting Step 1 accommodation by race, disability type, timing of diagnosis, and institutional resources.

## Teaser

This cross-sectional study examines the associations between race and ethnicity, disability type, timing of disability diagnosis, and access to specialized disability resource professionals with accommodation requests and approvals on the US Medical Licensing Examination (USMLE) Step 1.

## Introduction

Between 2015 and 2022, the percentage of medical students self-identifying as having a disability increased by 119%, from 2.7% to 5.9%.^1,2^ Despite this growth, medical students with disabilities (MSWD), especially MSWD from underrepresented backgrounds, continue to face substantial barriers, including limited access to accommodations and insufficient support navigating the accommodations request process.^3,4,5^ These barriers restrict access to equitable training opportunities,^4,5,6,7^ exacerbate burnout,^8,9^ and contribute to experiences of negative learning environments.^5,9,10^ Lack of accommodations may lead to disparities in academic progress, including increased odds of taking a leave of absence, delayed graduation,^11,12^ lower rates of induction into medical honor societies,^13^ and challenges securing residencies.^14^ These challenges may be magnified for students from historically marginalized backgrounds who often face additional financial and systemic obstacles, such as discrimination, burnout, and limited access to mentors and resources.^15,16,17,18^

MSWD often encounter limited institutional resources in medical training, including a shortage of specialized disability resource professionals (DRPs).^4,5,6,19,20^ Specialized DRPs have additional training and expertise in medical education and are uniquely equipped to evaluate reasonable accommodations, interpret disability documentation, identify functional impairments, and assess barriers related to the US Medical Licensing Examination (USMLE) series.^19,20^ A critical part of their role is guiding students through the USMLE Step 1 accommodations process by helping them identify needed accommodations, drafting letters of support, reviewing personal statements, and reducing the administrative burden of assembling a cohesive application.^4,5,6,19,20,21,22^ Students have identified this support as essential to successfully applying for Step 1 accommodations.^4,5,21^ Moreover, research suggests that specialized DRP assistance may reduce the demand for additional institutional resources, such as tutoring, clerkship rescheduling, and off-cycle coursework, which often arise following Step 1 accommodation denials and delayed entry to the clinical phase of training.^23^ Despite these benefits, one 2021 study^6^ found that only 9% of US medical schools employ specialized DRPs, leaving many MSWD to navigate a complex and high-stakes system with limited guidance.

The USMLE Step 1 is the first of 3 licensing examinations administered by the National Board of Medical Examiners (NBME) that all physicians must pass before becoming eligible for a US medical license. Overall, MSWD tend to score lower on Step 1 than their peers without disability. In one study,^11^ MSWD who were approved for accommodations on the NBME scored higher (on average, 5.9 points) than those without accommodations.

The NBME determines eligibility for accommodations under the Americans with Disabilities Act^24^; however, the process is often perceived as burdensome^25,26,27^ and unsuccessful.^23^ One study^25^ found that the NBME denied more than 50% of Step 1 accommodation requests, greatly disrupting medical training and, in some cases, forcing students to take leaves of absence to appeal the decision.

USMLE Step 1 performance is a key determinant of career progression. Despite its importance, there is limited understanding of how sociodemographic characteristics and institutional support influence the accommodation request and approval process. In this study, we examined the association of students’ gender, race, ethnicity, disability status and type, timing of diagnosis, and access to specialized DRPs with both the likelihood of requesting accommodations and the likelihood of those accommodations being approved for the USMLE Step 1.

## Methods

Between April 2023 and April 2024, we conducted a cross-sectional study examining MSWD USMLE Step 1 accommodation requests and approvals. This study adds to a previous multisite study^11^ by exploring the intersection of race, ethnicity, and disability status. A consortium of 9 US MD-granting medical schools collaborated on this research. The University of Michigan institutional review board deemed this study exempt from review and the need for informed consent because the data were deidentified. This study was conducted and reported in accordance with the Strengthening the Reporting of Observational Studies in Epidemiology (STROBE) reporting guideline.

Deidentified data were obtained from individual student records in collaboration with school administrators, specialized DRPs, or designated staff. School representatives identified students who graduated between 2020 and 2023 and who had been registered with disability services and received accommodations at any point during their medical school enrollment, and provided official documentation of their disability. The data included race and ethnicity (as reported through the American Medical College Application Service [AMCAS] at the time of application), disability type, timing of disability diagnosis (before or after matriculation), and USMLE Step 1 accommodation request and approval status.

### Gender and Race and Ethnicity

Gender was self-reported on the AMCAS and included male and female only. Self-reported race and ethnicity data on the AMCAS were consolidated into 3 categories: Asian, underrepresented in medicine (URiM), and White. URiM was defined as identifying with any of the following groups: American Indian, Alaska Native, Black or African American, Hispanic, or Native Hawaiian or Pacific Islander, as reported on the AMCAS. Students reporting multiple races and/or ethnicity categories were assigned to their least represented category to capture underrepresentation. Students for whom race and ethnicity is unknown were categorized into an unknown group.

### Disability Type and Timing of Disability Diagnosis

In this study we built on the initial classification used in previously published studies^11^ (ie, trichotomized types of chronic health conditions, physical or sensory disabilities, and cognitive disabilities), but further disaggregated the cognitive disabilities type into learning disabilities, psychological disabilities, and attention-deficit/hyperactivity disorder (ADHD). We evaluated ADHD as a distinct disability type given the high prevalence of ADHD in our sample and the longstanding challenges associated with documenting ADHD for USMLE Step 1 accommodation requests—factors that may influence both request and approval rates.^28,29^ Overall findings were classified into the following verified types of disability: ADHD, psychological disabilities (eg, depression, bipolar disorder, and anxiety), chronic health conditions (eg, lupus, autoimmune disorder, and diabetes), physical and/or sensory disabilities (eg, deaf or hard of hearing, visual, and mobility disabilities), and cognitive or learning disabilities (eg, specific learning disabilities and traumatic brain injuries). The timing of disability diagnosis was categorized as either occurring before or after matriculation into medical school.

### Specialized Disability Support

We created a school-level indicator to differentiate between institutions with specialized (3 schools) and general (6 schools) DRPs. Specialized DRPs receive advanced training in medical school disability services, including how to assist students with USMLE Step 1 accommodation requests, understanding testing agency requirements, and supporting students through accommodation requests including interpreting complex evaluations, applying these data to justify accommodation need, and providing feedback on students’ personal statements.^19^ In contrast, general DRPs do not have advanced training in medical education or USMLE request processes.

### Outcomes

The primary outcomes of interest included whether MSWD requested accommodations for the USMLE Step 1 examination and the approval status of those requests. The majority of students reported their decision to school administrators or DRPs; however, since students are not required to disclose NBME accommodation decisions, and these data are not directly reported to schools, some outcome data were missing.

### Statistical Analysis

Demographic characteristics were summarized for the overall study sample. Associations between demographic characteristics with requesting for and approval of Step 1 accommodations were assessed using χ^2^ tests of independence. Fisher exact test was used when expected frequencies in contingency tables fell below 5. Statistical significance was determined using a 2-tailed threshold of *P* < .05. *P* values were adjusted using the Bonferroni method in situations where post hoc pairwise comparisons were made. Analyses were conducted using R statistical software version 4.4.2 (R Project for Statistical Computing).

## Results

Among 295 MSWD in the study cohort (189 female [64%]), 41 (15%) identified as Asian, 162 (59%) as White, 70 (26%) as URiM. In total, 105 students (36%) reported a psychological disability, and 167 (58%) received a disability diagnosis before medical school matriculation (Table 1). Among the 9 schools included in the study, 3 (33%) had specialized DRPs, 4 (44%) were private, 3 (33%) were from the Northeast, 3 (33%) from the Central region, 2 (22%) from the Southern region, and 1 (11%) from the Western region. The average rate of accommodation requests for USMLE Step 1 across all schools was 34% (range, 15%-46%). The average NBME approval rate for USMLE Step 1 accommodation requests across all schools was 62% (range, 13%-96%).

**Table 1.** Characteristics of the Study Cohort

Variable	Students, No. (%) (N = 295)
Race and ethnicity (n = 273)^a^	
Asian	41 (15)
White	162 (59)
Underrepresented in medicine^b^	70 (26)
Unknown^c^	22
Gender (n = 295)	
Female	189 (64)
Male	106 (36)
Disability type (n = 292)	
Attention-deficit/hyperactivity disorder	72 (25)
Chronic health conditions	51 (17)
Cognitive and/or learning	45 (15)
Physical and/or sensory	19 (7)
Psychological	105 (36)
Unknown^c^	3
Disability diagnosis history (n = 286)	
Diagnosis before medical school	167 (58)
Diagnosis during medical school	119 (42)
Unknown^c^	9
NBME accommodations requested, Step 1 (n = 295)	
Not requested	175 (59)
Requested	120 (41)
NBME accommodations, Step 1 (n = 97)	
Accommodated	67 (69)
Not accommodated	30 (31)
Unknown^c^	198
Specialized disability resource professional (n = 295)	
No	113 (38)
Yes	182 (62)
Region (n = 295)	
Central	142 (48)
Northeast	109 (37)
South	24 (8)
West	20 (7)
School type (n = 295)	
Private	137 (46)
Public	158 (54)

Abbreviation: NBME, National Board of Medical Examiners.

^a^ *P* = .14.

^b^ Defined as identifying with any of the following groups: American Indian, Alaska Native, Black or African American, Hispanic, or Native Hawaiian or Pacific Islander.

^c^ Unknown values were not included in calculations of percentages.

The initial sample consisted of 413 MSWD from 9 institutions. Students with unknown Step 1 accommodation request status (118 students [29%]) were excluded, resulting in 295 students (71%) for analysis. Compared with those included in the study, a lower proportion of excluded students were White (53 students [49%] vs 162 students [59%]), reported cognitive or learning disability (10 students [9%] vs 45 students [15%]), received a disability diagnosis before medical school matriculation (45 students [42%] vs 167 students [58%]), and attended a school with specialized DRPs (8 students [7%] vs 182 students [62%]).

### Requesting Accommodations

Among 295 MSWD in the cohort, 120 (41%) requested accommodations. There was no significant association between gender and request for Step 1 accommodations (41 male students [38%] vs 79 female students [42%]). A χ^2^ test of independence showed a significant association between race and ethnicity and requests for Step 1 accommodations. Post hoc pairwise comparisons showed the difference between request rates among White students (74 students [46%]) and Asian students (8 students [20%]) to be statistically significant. Pairwise comparisons between White students and URiM students were not statistically significant (Table 2).

**Table 2.** Characteristics of Students With Disabilities by Step 1 Accommodation Request Status

Student characteristics	Total No. of students (N = 295)	Students, No. (%)		*P* value	
		Requested (n = 120)	Not requested (n = 175)	χ^2^ or Fisher exact test	Bonferroni-adjusted pairwise comparison
Race and ethnicity (n = 273)					
Asian	41	8 (20)	33 (80)	.01	.01
White	162	74 (46)	88 (54)		Reference
Underrepresented in medicine^a^	70	27 (39)	43 (61)		.92
Unknown	22	11 (50)	11 (50)		NA
Gender (n = 295)					
Male	106	41 (38)	65 (62)	.56	NA
Female	189	79 (42)	110 (58)		NA
Disability type (n = 292)					
Attention-deficit/hyperactivity disorder	72	36 (50)	36 (50)	.002	.05
Chronic health condition	51	27 (53)	24 (47)		.04
Cognitive and/or learning	45	22 (49)	23 (51)		.12
Physical and/or sensory	19	3 (16)	16 (84)		>.99
Psychological	105	31 (30)	74 (70)		Reference
Unknown^b^	3	1	2		NA
Disability diagnosis history (n = 286)					
Diagnosis before medical school	167	88 (53)	79 (47)	<.001	NA
Diagnosis during medical school	119	30 (25)	89 (75)		NA
Unknown^b^	9	2	7		NA
School with specialized DRP (n = 295)^c^					
No (n = 6)	113	52 (46)	61 (54)	.14	NA
Yes (n = 3)	182	68 (37)	114 (63)		NA

Abbreviations: DRP, disability resource professional; NA, not applicable.

^a^ Defined as identifying with any of the following groups: American Indian, Alaska Native, Black or African American, Hispanic, or Native Hawaiian or Pacific Islander.

^b^ Unknown values were not included in calculations of percentages.

^c^ Specialized DRPs are DRPs specific to the medical school who have received advanced training in medical school accommodation processes, including supporting students’ requests for US Medical Licensing Examination Step 1 accommodations.

There was a significant association between disability type and request for accommodations. Compared with students with psychological disabilities (31 students [30%]), students with ADHD (36 students [50%]) and chronic health conditions (27 students [53%]) were more likely to request accommodations on USMLE Step 1. Additionally, the timing of disability diagnosis was significantly associated with requests for accommodations, with students who received a diagnosis before medical school more frequently requesting accommodations (88 students [53%]) than those who received a diagnosis during medical school (30 students [25%]).

We also examined the association of the presence of a specialized DRP with requests for accommodations. No significant associations were found between requests for Step 1 accommodations and attending a school with a specialized DRP (Table 2).

### Approval of Accommodation Requests

Among 120 MSWD who applied for Step 1 accommodations, 23 (19%) had missing approval data and were excluded. Excluded and included groups were demographically similar, with no significant difference by race and ethnicity, gender, and disability type. Of the remaining 97 MSWD, 67 were approved for accommodation, resulting in an overall approval rate of 69%. There was no significant association between gender and approval of Step 1 accommodation requests (22 male students [71%] vs 45 female students [68%]). Among 87 students with known race and ethnicity, there was no statistically significant association between race and ethnicity with approval rates (41 White students [68%], 4 Asian students [67%], and 14 URiM students [67%]).

Approval rates significantly differed across disability categories. The lowest accommodation approval rates were found among medical students with psychological disabilities (14 students [52%]) and ADHD (13 students [52%]). In post hoc analysis, compared with medical students with psychological disability, students with chronic health disability were significantly more likely to have USMLE Step 1 accommodations approval (Table 3). No significant differences were found for other disability types.

**Table 3.** Characteristics of Students With Disabilities by Step 1 Accommodation Approval Status

Characteristics	Total No. of students (N = 97)	Students, No. (%)		*P* value	
		Accommodated (n = 67)	Not accommodated (n = 30)	χ^2^ or Fisher exact test	Bonferroni-adjusted pairwise test
Race and ethnicity (n = 87)					
Asian	6	4 (67)	2 (33)	>.99	>.99
White	60	41 (68)	19 (32)		Reference
Underrepresented in medicine^a^	21	14 (67)	7 (33)		>.99
Unknown^b^	10	8	2		NA
Gender (n = 97)					
Male	31	22 (71)	9 (29)	.82	NA
Female	66	45 (68)	21 (32)		NA
Disability type (n = 96)					
Attention-deficit/hyperactivity disorder	25	13 (52)	12 (48)	.002	>.99
Chronic health condition	24	22 (92)	2 (8.3)		.02
Cognitive and/or learning	19	16 (84)	3 (16)		.12
Physical and/or sensory	1	1 (100)	0		>.99
Psychological	27	14 (52)	13 (48)		Reference
Unknown^b^	1	1	0		NA
Disability diagnosis history (n = 96)					
Diagnosis before medical school	75	57 (76)	18 (24)	.004	NA
Diagnosis during medical school	21	9 (43)	12 (57)		NA
Unknown^b^	1	1	0		NA
Schools with specialized DRP (n = 97)^c^					
No (n = 6)	34	18 (53)	16 (47)	.01	NA
Yes (n = 3)	63	49 (78)	14 (22)		NA

Abbreviations: DRP, disability resource professional; NA, not applicable.

^a^ Defined as identifying with any of the following groups: American Indian, Alaska Native, Black or African American, Hispanic, or Native Hawaiian or Pacific Islander.

^b^ Unknown values were not included in calculations of percentages.

^c^ Specialized DRPs are DRPs specific to the medical school who have received advanced training in medical school accommodation processes, including supporting students’ requests for US Medical Licensing Examination Step 1 accommodations.

The Step 1 approval rate also significantly differed by the timing of disability diagnosis. Among 96 students with known timing of disability diagnosis, MSWD with a diagnosis before medical school had higher approval rates (57 students [76%]) than those who received a diagnosis in medical school (9 students [43%]). In addition, MSWD at schools with a specialized DRP had higher approval rates (49 students [78%]) compared with those at schools without a specialized DRP (18 students [53%]) (Table 3).

## Discussion

The findings of this cross-sectional study suggest that although approval rates in this sample were higher than those reported in prior studies,^11,25^ disparities in access to USMLE Step 1 accommodations still persist. Asian MSWD, medical students with psychological disabilities, and those receiving a disability diagnosis after matriculation face disparate outcomes. Among these groups, medical students with psychological disabilities and those receiving a diagnosis after entering medical school had the lowest approval rates. Strikingly, there was wide variation between the percentage of medical students who request accommodations and the approval rates across institutions, with higher approval rates at medical schools employing a specialized DRP.

In this study, we found barriers to Step 1 accommodation requests and approvals. The low rates of requests among Asian medical students may reflect uncertainty of disability diagnosis and broader systemic barriers, such as delayed access to early diagnosis, financial constraints, or insufficient institutional support.^3,30,31,32^ The cultural stigma surrounding disabilities or learning challenges may also discourage students from pursuing disability diagnosis and acknowledging the need for accommodations. As a result, many Asian students may choose to push through challenges rather than request the assistance to which they are entitled. The low request rates may also reflect a global belief system about the probability of receiving accommodations given specific scenarios (eg, diagnosis and history of accommodation).^5,23,29^ Finally, success after litigation may further spur the belief that the only viable pathway to accommodation is through legal action, which comes with great psychological toll.^27^

Significantly lower accommodation approval rates were noted for students with cognitive and/or learning disabilities. Accommodation denials often stem from a lack of early diagnosis and the prohibitively high cost of neuropsychological testing.^33^ In our sample, MSWDs from schools without specialized DRPs and those receiving a disability diagnosis during medical school experienced particularly low approval rates for Step 1 accommodations. Accrediting bodies for US medical schools—the Liaison Committee on Medical Education^34^ and the Commission on Osteopathic College Accreditation^35^—have a unique opportunity to help reduce barriers faced by MSWD by strengthening their expectations related to disability support. Currently, accreditation requirements specific to MSWD are limited to expecting medical schools to develop and publish technical standards outlining the abilities necessary to meet program requirements, with or without accommodations.^35,36^ These accrediting bodies might consider requiring institutions to ensure access to specialized DRPs, thereby streamlining the accommodations process by providing expertise in navigating a complex and highly individualized process while also helping to eliminate potential conflicts of interest by ensuring that accommodation decisions are made by neutral professionals rather than faculty or administrators involved in student evaluation.^6^ For schools with limited financial resources, sharing a specialized DRP with another program, such as a nursing or physician associate program, or hiring a specialized DRP at a per diem or consultation capacity may be practical alternatives to employing a full-time specialized DRP.

Medical schools can implement strategies that prioritize early identification of disability-related barriers, equitable support for MSWD, and effective communication about how to request accommodation. These strategies may include outreach initiatives and opportunities for low or no-cost screening and neuropsychological testing, and broad and frequent dissemination of resources. By normalizing discussions about disabilities, schools can foster a supportive environment where MSWD feel empowered to seek accommodations without fear of stigma. Medical schools might offer a vetted list of culturally humble diagnosticians, who are familiar with systemic barriers faced by MSWD and the NBME documentation standards, to improve diagnostic accuracy and equity for Asian and URiM MSWD.^37^

For MSWD the accommodation request process can be a tumultuous period, necessitating a leave of absence, which may provide the necessary flexibility to navigate the Step 1 accommodations process. However, given the consequences of taking a leave of absence (eg, extended time to graduation and lower placement rate into residency),^38,39,40^ medical schools may wish to consider developing mechanisms to maintain student enrollment while also providing wrap-around support for navigating accommodation requests and the appeals process.

The NBME plays a critical role in addressing structural and cultural barriers to accommodations within their examinations. Simplifying documentation requirements, providing clear application instructions, and ensuring staff are trained on implicit bias and ableism are essential to creating a more equitable review process.

The NBME should consider further examining disparities in accommodation approvals for MSWD who receive a diagnosis after matriculating into medical school. Many of these students successfully used compensatory strategies throughout their earlier education but may find these strategies inadequate in medical school’s demanding environment. Disparity in accommodations based on the timing of diagnosis can unfairly disadvantage otherwise qualified students. Additionally, the current 60-business-day turnaround for accommodation decisions can greatly delay students’ progression. The NBME could aim to reduce the timeline to decision and encourage medical schools to provide MSWD with early, clear guidance on documentation and deadlines. These actions may prevent unnecessary delays and stressors associated with the application process.

Although we found that the NBME approved 69% of Step 1 accommodation requests, persistent and inaccurate rumors exist that suggest accommodations are rarely granted. The perpetuation of these rumors can be harmful to MSWD. MSWD who believe these myths may refrain from applying for accommodations, ultimately preventing the NBME from accurately capturing the prevalence and needs of MSWD. Although the NBME recently published high level request and approval rates,^41^ these data are not disaggregated by disability type, by whether accommodations were fully or partially approved, and the time to decision, which does not allow for a full evaluation of their processes. Providing detailed data may dispel misinformation, reduce fear of denial, and help identify disparities in accommodation across disability types. Although this study focused on MSWD seeking disability accommodations from the NBME for the USMLE Step 1 examination, these recommendations would be beneficial in other contexts involving standardized achievement or licensing examinations, including the Comprehensive Osteopathic Medical Licensing Examination of the US, which is administered by the National Board of Osteopathic Medical Examiners (NBOME). In line with efforts in Canada^42^ and the United Kingdom,^43^ the NBME and NBOME should also consider collaborating with researchers, medical associations, and disability advocacy groups to produce scholarship on accommodation practices, further advancing transparency, accountability, and promoting equity.

Finally, if not already in place, the NBME and NBOME might establish an advisory committee composed of diverse community members, including specialized DRPs, MSWD, student affairs deans, and experts in high-stakes testing bias. Such a committee could also inform ways to streamline the accommodations process, improve decision timelines, enhance transparency, and build trust while reducing the psychological and logistical burdens placed on MSWD.

### Limitations

To our knowledge, this study provides the most comprehensive data to date on USMLE Step 1 accommodations requests and approvals for MSWD, but several limitations must be acknowledged. First, the graduate-only sample risks survivor bias, excluding students who may have left due to accommodation challenges, including nonaccommodation and subsequent failure of Step 1 and dismissal or withdrawal. Missing data—unknown request status for 29% of students and unknown approval status for 19%—limit accuracy, especially in schools without specialized DRPs and in the 3 schools with over 70% unknown request data. Schools with specialized DRPs may inflate rates and reflect a best case scenario because of stronger disability support. We could not assess school policies, cultures, or quality of disability services. These factors limit generalizability and intersectional analysis, highlighting the need for broader studies and additional data from NBME and NBOME.

Second, the study included primary disability diagnoses, limiting insight into students with multiple disabilities. It also does not fully address reasons for nonrequest, the effects of Step 1 going pass/fail, or barriers like stigma, limited awareness, and complex processes.^44^ For students who received a diagnosis during medical school, timing relative to Step 1 is unclear, complicating analysis. Binary approval data may also include partial approvals, limiting understanding of full or partial access to Step 1 examination accommodation. Despite these limits, the study provides novel insights into potential inequities in accommodation requests and approvals and actionable recommendations.

## Conclusion

Findings from this initial evaluation reveal systemic inequities in USMLE Step 1 accommodation requests and approvals. Improving transparency, streamlining applications, and providing specialized DRP support—especially for marginalized students and those who receive a diagnosis during medical school—may reduce these gaps. Although this study is small, it offers the most comprehensive analysis to date, highlighting the need for more data and coordinated action from accrediting bodies, licensing organizations, and schools to ensure equitable access to accommodations.

## Notes

Supplement.
Data Sharing Statement
