# Supplementary material for: Race and Disability Characteristics and Accommodation Disparities on the USMLE Step 1
Source: JAMA Netw Open. 2025 Sep 30;8(9):e2534621. doi: 10.1001/jamanetworkopen.2025.34621 (PMC12485634; doi:10.1001/jamanetworkopen.2025.34621)
Supplement: Supplement. — Data Sharing Statement [file jamanetwopen-e2534621-s001.pdf]

Data Sharing Statement

Nguyen. Race and Disability Characteristics and Accommodation Disparities on the USMLE Step 1. *JAMA Netw Open*. Published September 30, 2025.
doi:10.1001/jamanetworkopen.2025.34621

Data
Data available: No
